# Distinct X-chromosome SNVs from some sporadic AD samples

**DOI:** 10.1038/srep18012

**Published:** 2015-12-09

**Authors:** A. Gómez-Ramos, P. Podlesniy, E. Soriano, J. Avila

**Affiliations:** 1 Centro de Investigación Biomédica en Red de Enfermedades Neurodegenerativas (CIBERNED), ISCIII, Madrid 28031, Spain; 2 Centro de Biología Molecular Severo Ochoa (CSIC-UAM), Neurobiology Laboratory, Madrid 28049, Spain; 3 Neurobiology Unit, Institut d’Investigacions Biomèdiques de Barcelona, CSIC, IDIBAPS, Barcelona 08036, Spain; 4 Department of Cell Biology, Faculty of Biology, University of Barcelona and Parc Científic de Barcelona, Barcelona 08028, Spain; 5 Vall d’Hebrón Institut de Recerca (VHIR), Barcelona 08023, Spain; 6 ICREA Academia, Barcelona 08010, Spain

## Abstract

Sporadic Alzheimer disease (SAD) is the most prevalent neurodegenerative disorder. With the development of new generation DNA sequencing technologies, additional genetic risk factors have been described. Here we used various methods to process DNA sequencing data in order to gain further insight into this important disease. We have sequenced the exomes of brain samples from SAD patients and non-demented controls. Using either method, we found a higher number of single nucleotide variants (SNVs), from SAD patients, in genes present at the X chromosome. Using the most stringent method, we validated these variants by Sanger sequencing. Two of these gene variants, were found in loci related to the ubiquitin pathway (*UBE2NL* and *ATXN3L*), previously do not described as genetic risk factors for SAD.

Alzheimer disease (AD) has been classified mainly into two types, namely early or late-onset AD (EOD or LOAD). In the former, the disease appears in the fifth or sixth decade of life whereas in the latter it usually appears after 70 years of age. EOD cases are related to family inheritance of *APP, PSEN1* and *PSEN2* genes mutated at specific sites^1^. This type of AD is known as Familial Alzheimer Disease (FAD) and it accounts for only about 1% of total cases^2^. However, most of the remaining cases, LOAD, also known as Sporadic Alzheimer Disease (SAD), are of unknown origin, aging being the main risk for the disease. In addition, several environmental and genetic factors have been proposed as risk factors for SAD. It has been reported that the presence of variants in several susceptibility loci increases the probability of developing the disease^3^. Among these, the variant 4 of *APOE* is the most prevalent genetic risk factor described to date^4^. Also, other genome-wide genes associated with SAD, have been linked not only to cholesterol metabolism, like *APOE*, but to other pathways related to immunity and endocytosis^5^. In addition, it has also recently been proposed that somatic gene variations acting on specific brain cells could be involved in the onset of the disease^6^. In this case, and in contrast to FAD, the variations are present only in neuronal tissues, being absent in peripheral ones^6^. These variations may arise from somatic mutations that take place during the development (or during adulthood) of the patient’s organism. Another study showed that the appearance of SNVs is not random throughout the genome but that there is a higher proportion of these variants at specific chromosomes^7^. The analysis in that study was done using a method that allows the differentiation of specific SNVs in a small population of cells in a given sample. However, the presence of other cells types lacking the SNV in the same sample can hinder the validation of these variants using other sequencing analyses like Sanger’s method. Thus, here, we used an alternative more stringent method to analyze DNA sequencing data from brain samples from SAD patients and non-demented controls.

Using the data obtained by Illumina technology sequencing, here we have applied three methods to process DNA sequencing data from brain exome samples, with the aim to identify the most suitable method for a further classical validation, using the Sanger method. A comparison of the results of the three methods has revealed a broad correlation among them as the three ones focused on the presence of SNVs specific to exomes of SAD patients located at specific chromosomes. Interestingly, we found an increased proportion of these SNVs in the X chromosome, a chromosome linked to some brain diseases^8^,^9^,^10^. Also, using the strictest of the three methods, the variations identified by Illumina technology sequencing have allowed successful validation by the Sanger method. Among these SNVs were those present at the X chromosome and located at *ATXN3L* and *UBE2NL*, two genes involved in the ubiquitin pathway^11^.

## Results

### The proportion of the number of SNVs from SAD patients varies depending on their chromosome localization

**Figure 1:**
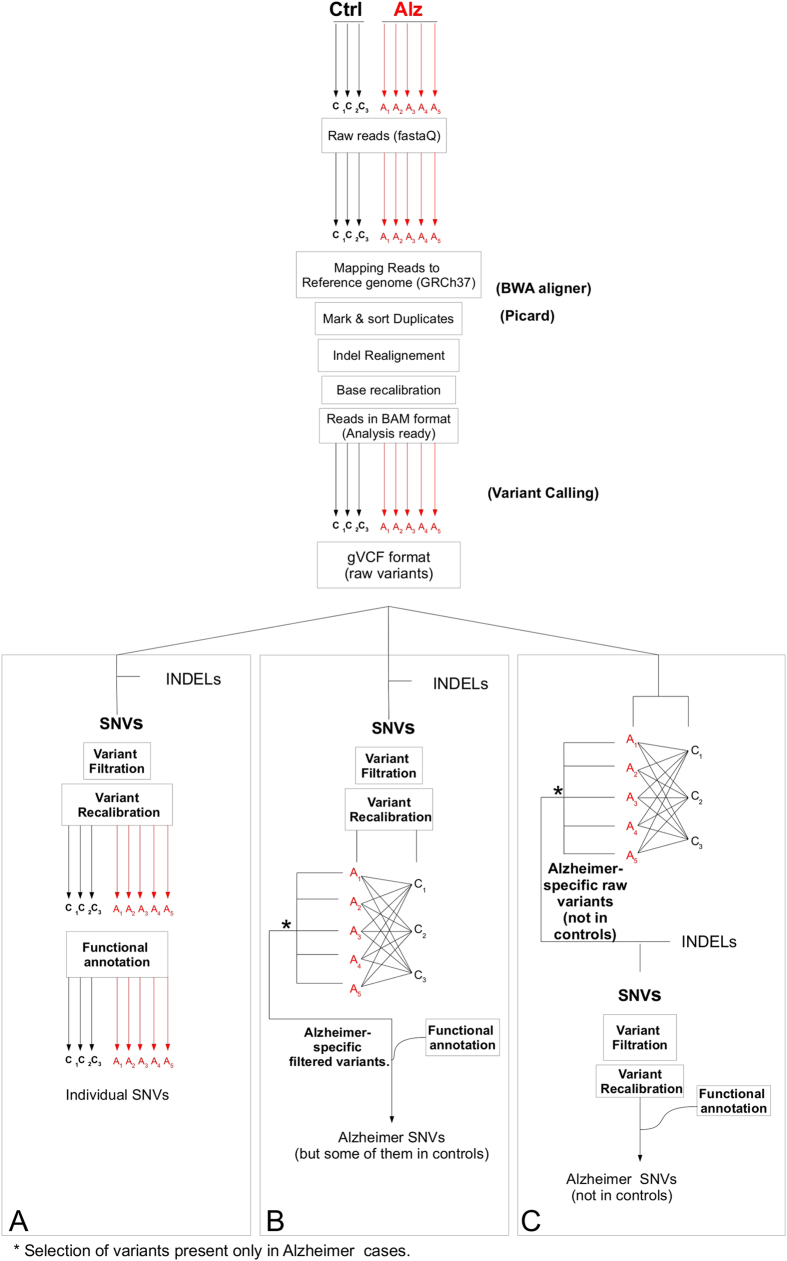
Number of exomic brain SNVs and their chromosomal distribution in sporadic Alzheimer disease patients and control subjects. (**A**) Chart showing the percentage of SNVs for each chromosome in samples from non-demented controls (C1–C3) and sporadic Alzheimer disease (SAD) patients (A1–A5). (**B**) The graphic shows the average percentage of SNVs found in the hippocampus for each chromosome for three non-demented control (**C**) and five SAD (**A**) samples. A significant difference in the number of SNVs was found for the X chromosome (inset). Error bars show standard deviation in each case and double asterisk is statistically significant (P < 0.001) compared with control cases. (**C**) Plot showing the difference between the average of the number of exonic SNVs per chromosome in samples from SAD patients and from non-demented controls.

Hippocampal exome sequencing of two male and three female SAD patients and 3 male non-demented controls, analyzed by method A (see methods), indicated the presence of specific SNVs. Thus, we studied the number of these SNVs present in the chromosomes of the eight subjects. Figure 1A shows a similar, but not identical pattern of SNVs per chromosome in the three non-demented controls (C1–C3). Also, for SAD (A1–A5) patients, a similar but not identical pattern of distribution of the SNVs along the chromosomes was found.

In a further step, we compared the average number of SNVs per chromosome in SAD patients and non-demented controls (Fig. 1B). Higher numbers of SNVs at the X chromosome were detected in the former (Fig. 1B, inset).

The difference in the number of SNVs in the X chromosome of SAD cases compared to controls may be attributable to the disease; however, it could also result from the gender of the donors. In order to check this, we performed a similar analysis to the previous one, testing the number of exonic SNVs per chromosome in the samples. However, on this occasion we treated the samples discriminated by gender. Supplementary Figure 1A shows the difference in SNV distribution along the chromosomes for the average of all the samples from male SAD cases. A very similar distribution to that observed in the previous case, in Fig. 1B, was detected, with a significant difference in the number of SNVs for the X chromosome in SAD samples (Suppl. Fig. 1A, inset). No such difference was detected when we compared the average number of SNVs per chromosome in for all the SAD samples grouped by gender (Suppl. Fig. 1B). No significant differences in the number of SNVs were appreciated for the X chromosome (Suppl. Fig. 1B, inset). When another two non-demented controls (C4 and C5, see Table 1) were tested, no differences were found between this additional control and the rest of them, having not SNVs in the loci in where Alzheimer-SNVs were found (see results for chromosome X in Supplementary Figure 4B and Supplementary Table 1).

### Chromosomal distribution of SNVs present only in SAD samples, using the three methods

**Figure 2:**
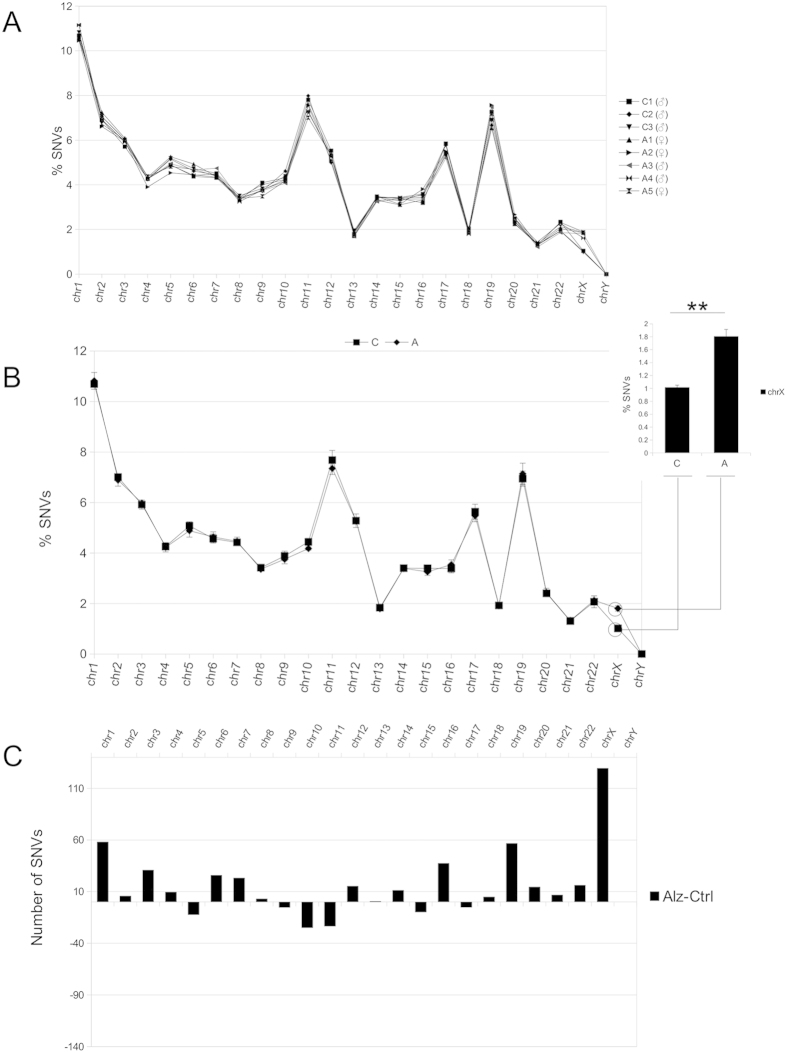
Methods used to determine SNVs. Representation of the three methods used to analyze individual and SAD-specific SNVs (present in sporadic Alzheimer disease (SAD) samples but not in non-demented controls). The three methods begin with the alignment of raw reads in FastaQ format and end with the raw data containing SNVs achieved after the variant calling process. From here, we used three alternative approaches to obtain individual or SAD-specific SNVs.(**A**) Scheme showing the method used to obtain individual exonic SNVs in the SAD (A1–A5) or non-demented control (C_1_–C_3_) samples (see methods). (**B**,**C**) Methods used to determine the presence of SAD-specific SNVs. The difference between the two methods is that in B the selection of SAD-specific SNVs was done after filtering variants using specific parameters (see methods) while in C this selection was done beforehand, using raw data obtained from variant calling. Method C proved more exact for the validation of the SNVs obtained by Sanger’s sequencing (see results). For a more detailed explanation, see methods.

To identify the SNVs that could be related to SAD, we searched for those SNVs present in the exomes of SAD cases but not in non-demented controls. For this purpose, using a comparative analysis, we proceeded in two ways to determine those SNVs present only in SAD samples. In the first case (method B, Fig. 2B), we selected only those SNVs present in SAD samples after a previous selection of the variants that passed the hard filter parameters suggested by software developers (http://gatkforums.broadinstitute.org/discussion/2806/howto-apply-hard-filters-to-a-call-set, see methods). In the second approach, we obtained SAD-specific SNVs (method C, Fig. 2C) by analyzing the files containing the variants at a previous level, in such a way that the selection of SAD-specific SNVs was done before filtering, comparing the raw files containing the SNVs obtained after calling the variants. Having obtained the SAD-specific raw data, we continued in a similar way to method B. We chose to do the comparison at this level of the analysis rather than to proceed comparing the files after filtering in order to avoid false negatives. Thus, when we have compared the files containing SNVs at a raw level and selected only the data containing variants present in SAD files, we can ensure that these selected variants were exclusive to the disease, and if these selected data have not enough quality or are not real SNVs, they will not pass the subsequent process of selection of real SNVs (filtering), indicated in methods.

**Figure 3:**
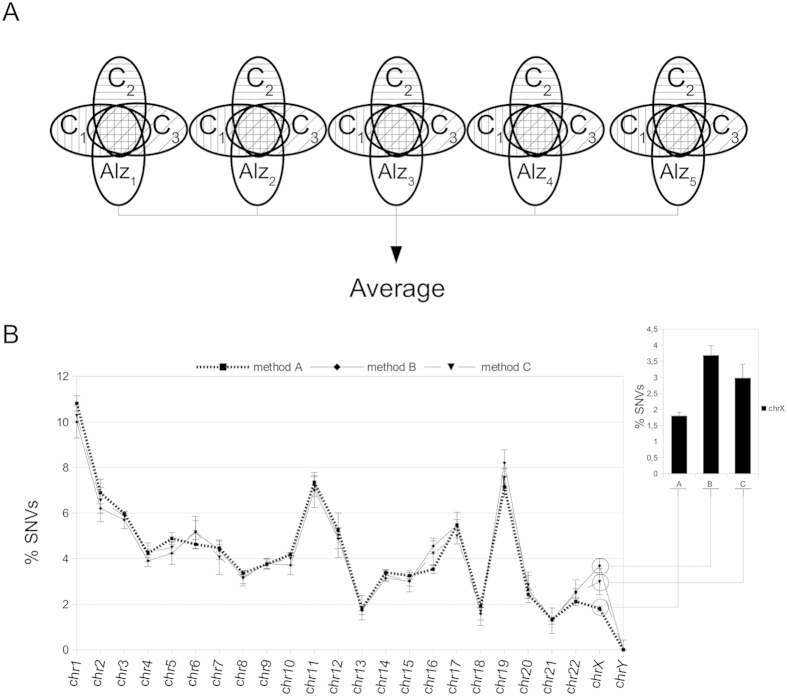
Number of SNVs present in sporadic Alzheimer disease patients and their chromosomal distribution. (**A**) Venn diagrams showing the part of the files containing SNVs considered specific to SAD samples. We selected those SNVs present only in SAD samples, which we refer to as Alz_1_–Alz_5_. (**B**) The graphic shows the average percentage of SNVs per chromosome from the five previously described samples of hippocampal exonic SAD-specific SNVs in Fig. 3A (Alz_1_–Alz_5_), obtained by methods B and C (described in Fig. 2B,C respectively). Also, the average of the number hippocampal exonic SNVs per chromosome obtained by method A (present in all SAD samples, but not necessarily exclusive to them) (described in Fig. 1B) is shown in order to facilitate comparison of the profiles obtained by the three methods. The inset included in this figure shows the values obtained for the X chromosome by the three methods. Note that the proportion of SNVs for this chromosome is even larger when the variants are SAD-specific (method B and C) than when they are present in but not exclusive to SAD samples (method A).

To compare the pattern of SAD-specific SNVs with those variants in all the SAD samples but not necessarily exclusive to this disease (method A), we plotted the chromosomal distribution of these variants for these three methods (Fig. 3B). We found a similar chromosomal distribution in the case of the SAD-specific SNVs (obtained by methods B and C) as well as those present in SAD samples but not exclusive to the disease (method A) (Fig. 3B). The proportion of SNVs for the X chromosome was higher for methods B and C than for method A (Fig. 3B, inset).

### Chromosomal distribution of the SNVs common to all SAD samples, using methods B and C

**Figure 4:**
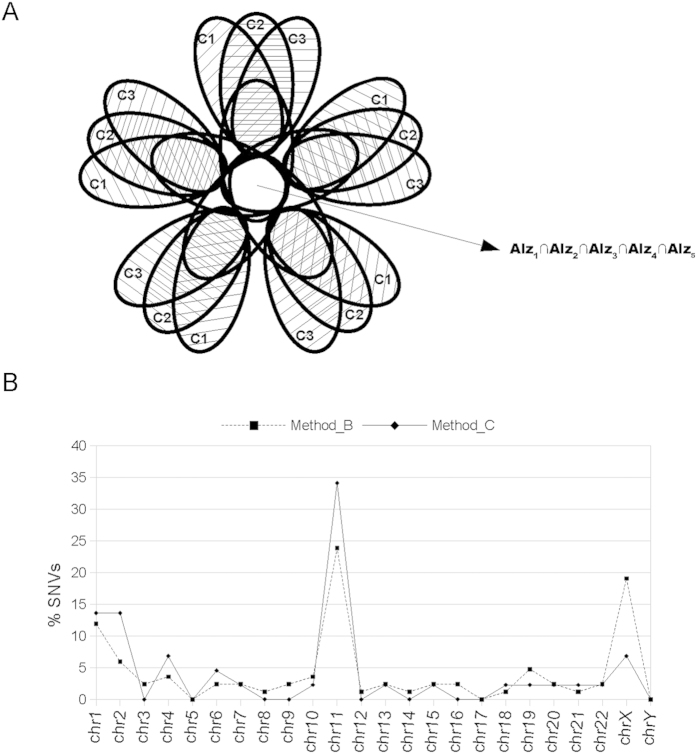
Chromosomal distribution of specific SNVs common to all samples from patients with sporadic Alzheimer disease. (**A**) In a similar way to that described in Fig. 3A, this Venn diagrams shows the part of the SNVs specific to sporadic Alzheimer disease (SAD) represented in Fig. 4B. We selected those hippocampal SNVs common to all SAD samples but absent in the controls. (**B**) This plot represents the distribution of the percentage of specific SNVs per chromosome common to all SAD samples (represented in previous Fig. 3A as Alz 1∩2∩3∩4∩5) obtained by methods B and C (see text and methods).

In order to identify the SNVs specific to SAD, we chose the intersection set of variants common to all the files containing SNVs present in SAD cases but not in any of the controls (Fig. 4A). We proceeded in this way for both method B and C and calculated the number of SNVs for each chromosome in a similar way to previous figures. The SNV distribution pattern per chromosome varied in most cases (Fig. 4B). The proportion of SNVs per chromosome was higher in some cases, such as for the X chromosome and chromosome 19, SNVs found in chromosome 11 varied in the opposite way. Taking into account that the X chromosome showed a higher proportion of SNVs in samples from SAD patients than in those from non-demented controls, we examined the characteristics of the variants specific to the former and the corresponding genes in which they were found. Using methods B and C, we obtained a total of 84 and 42 SNVs along all the chromosomes, respectively. All the SNVs detected by method C were also included in those identified by method B (Table 2).

### Characteristics of the genes bearing SNVs exclusive to SAD samples and located at the X chromosome

With respect to the SNVs found by means of method C, the most stringent approach, we detected 42 exonic SNVs exclusive to SAD samples and therefore not present in any controls (see Table 2, in bold). Three of these SNVs were in loci present in the X chromosome, namely in *ATXN3L* (putative ataxin-3-like protein), *COL4A6* (collagen type IV alpha 6), and *UBE2NL* (ubiquitin-conjugating enzyme E2N-like). The variation in UBE2NL adds a stop signal to the DNA sequence, causing a truncation in the protein (L89*). This alteration may affect the function of this gene. The variation in the COL4A6 sequence does not affect translation because it is a synonym change, while the variation in the locus of ATXN3L involves the replacement of a glycine by an aspartate residue in position 332 of the protein. Looking at Gene Ontologies, the two genes with SNVs in the X chromosome have biological processes annotations related to the ubiquitin-proteosome system. Therefore, ATXN3L is involved in protein de-ubiquitination^11^ while UBE2NL is an ubiquitin-conjugating enzyme^12^.We have used hippocampal samples to have both somatic and germiline variations. In some cases (when available), we have already sequenced blood samples. The comparison of neuronal with these non-neuronal tissues showed a very similar pattern for the studied SNVs, being almost identical in both (see a representative result in Supplementary Figure 3). This result indicates that, at least in these cases, the SNVs have a germinal origin; this also could explain why some changes, sometimes, occur in non-neuronal tissues, during AD development^13^.

### Validation of the SNVs specific to the exomes of SAD patients by a) alignment of the reads and b) Sanger sequencing

**Figure 5:**
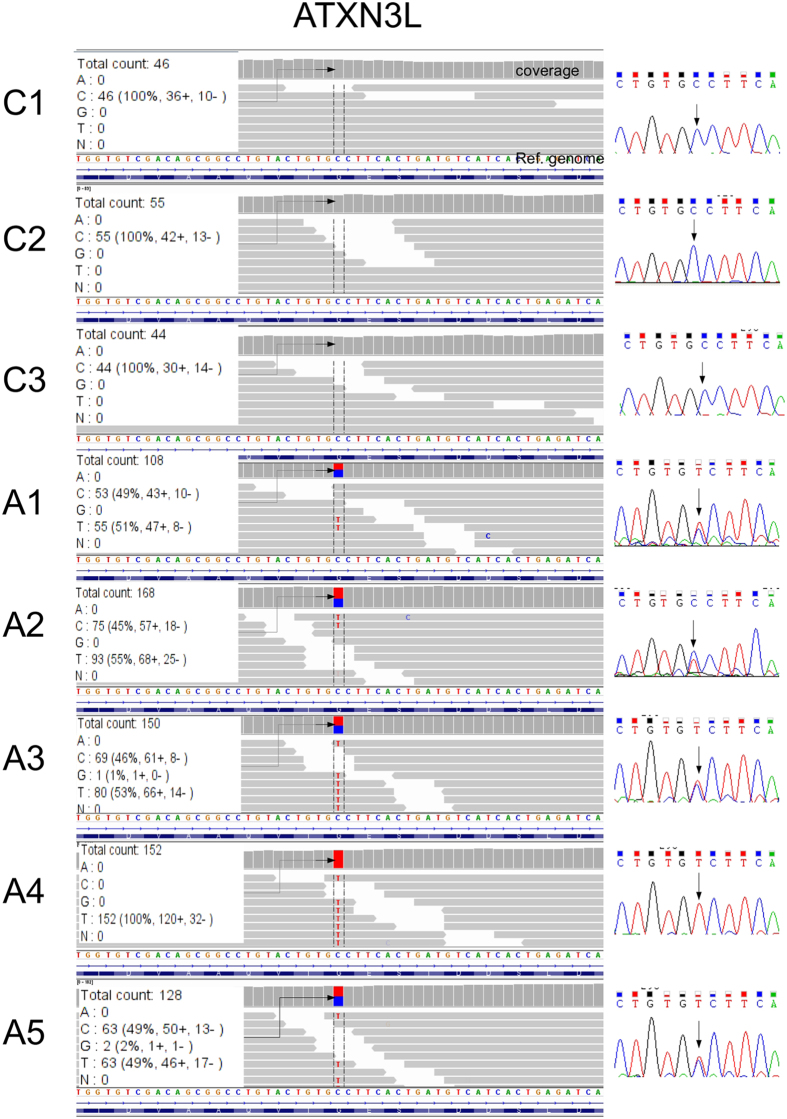
Example of validation by Sanger’s sequencing of a SNV specific to sporadic Alzheimer disease and found in the X chromosome by method C. This figure shows a representative example of the read coverage (left part) and Sanger’s sequencing (right part) for a representative locus found in the X chromosome, in *ATXN3L*, by method C, an approach used to determine SNVs specific to sporadic Alzheimer disease (see methods). The results obtained by Sanger’s sequencing validate in every case both the presence of the SNV and its genotype (homozygous or heterozygous). C1–C3 correspond to non-demented controls and A1–A5 correspond to AD patients.

A further validation was done by Sanger’s sequencing, comparing exome regions of SAD patients with those of non-demented controls. Figure 5 shows a representative validation of the data obtained by method C. All the regions surrounding the SNVs that we checked showed a correct correlation between the alignments of the reads against the reference genome and with the results obtained by Sanger sequencing, in all cases being homozygous or heterozygous. Thus, the validation of the SNVs by Sanger sequencing was achieved only by method C (those that are in bold in Table 2, see supplementary Figure 3). Using method B, we found that, in most cases, the lack of alignments of the reads surrounding the SNVs resulted in false negatives in the control samples (not shown). This observation could be attributed to the fact that in this method the comparison between the files containing SNVs was performed after a strict filtering of the reads on the basis of their quality. This filtering procedure used in method B ensured high quality of the SNVs obtained. However, the validation tests by Sanger sequencing and alignments show that some of the SNVs isolated by this approach are not always specific to SAD, but they are also present in some non-demented controls (not shown). On the other hand, when the comparison between files containing SNVs was done at a previous level, before filtering (like method C), and the loci considered as SAD-specific are then filtered using the same criteria, we can expect to obtain high quality SAD-specific SNVs that can be validated by Sanger sequencing.

In all the cases (method C), we found a correct correlation between the loci checked by Sanger sequencing and those expected to correspond by alignment (Fig. 5).

## Discussion

Here we compared three methods to analyze the DNA sequences of hippocampal exomes from SAD patients and non-demented controls. We examined the number of SAD-specific SNVs in the chromosomes. Method A (see methods) yielded a descriptive catalog of all the SNVs present in the chromosomes, following the recommended workflows for variant analysis using the software included in GATK (https://www.broadinstitute.org/gatk/guide/best-practices). This approach is commonly used to obtain exonic SNVs; however, it does not indicate differences between exomes. Method B has been used in a similar way^6^ to determine somatic mutations present in neuronal but not in peripheral tissues of a single SAD patient. A more strict approach, method C, described in the present study, allowed us to identify SNVs exclusive to SAD patients by means of an alignment step, and to later validate them by Sanger sequencing. The description of this method C is probably one of the strengths of the present study; the limitation of our study could be related to the small sample size, which might reduce the statistical significance of our work.

In this study we used brain tissue, since in these samples we can not only identify variants that could be inherited but also we can look at the presence of somatic mutations present in neuronal but not in peripheral tissue. Our results reveal a major difference in SAD-specific SNVs present in the X chromosome. Curiously, this chromosome contains an excess of genes that are highly expressed in brain tissue^8^,^14^,^15^.

Also, variations in genes present in chromosome X have been related to some neuronal diseases. In this way, some genes present in the X chromosome, have been reported to participate in brain function and dysfunction mainly of X-linked forms of mental retardation^8^. Moreover, a higher number of cognition genes have been identified in the X chromosome than in comparable-length segments of autosomes^8^,^10^

The higher increase in chromosome X found for SNVs of SAD patients, and the fact that males have one copy of the X chromosome while females have two, could make males more susceptible to SAD. However, this notion is not supported by current data^2^, which indicate a higher prevalence of SAD among females. This discrepancy could be explained by other non-genetic risk factors balancing out^16^ the possible prevalence of X-linked risk factors in males.

Furthermore, the distribution of SAD-specific SNVs in the X chromosome appears to be random (supplementary Figure 2).

*COL4A6*, *ATXN3L* and *UBE2NL* were among the genes in the X chromosome showing SNVs present in all the DNA samples from SAD patients. These three genes are expressed in the brain and they have functions that could be related to SAD pathology. We compared the present data with previous loci detected in GWAS studies., We found that a SNV at ATXN3L, locus chrx:13337059, has been already reported (http://www.gwascentral.org/), whereas for COL4A6, no correlation was found with the previous 66 reported SNVs. This is compatible with the possibility that the SNVs described herein may arise from somatic mutations, although further analyses are required to confirm this possibility. For the 4BE2NL gene, no GWAs data are available. In relationship with SNVs resulting from somatic changes in AD, there are two recent studies reporting the presence of low allele frequency mosaic mutations in the brain of AD patients, both of them focused to scrutinize SNVs at the APP, PSEN1, PSEN2 and MAPT loci^17^,^18^.

*COL4A6* encodes one of the six subunits of type IV collagen. The amount of this collagen is significantly increased in the cerebral micro-vessels of subjects with SAD compared to age-matched controls^19^. Another type of collagen, collagen VI, protects neurons against Abeta toxicity^20^.

ATXN3L is a deubiquitinating enzyme expressed in brain and associated with Machado-Joseph disease^21^; however, whether this protein is related to SAD remains unclear. UBE2NL is another protein related to the ubiquitination-de-ubiquitination process. It is also expressed in brain and it participates in parkin-dependent mitophagy^22^, a process that can be dysregulated in AD.

Finally, we do not know whether these changes in ubiquitination-de-ubiquitination affect tau, a key protein in SAD pathology that can undergo ubiquitination as a post-translational modification^23^,^24^. Interestingly, using method B, we identified another gene, *USP51*, also related to ubiquitination, in all the SAD samples tested.

In summary, here we describe a new method to identify SNVs from exome DNA sequencing by Illumina techniques. These SNVs can be later validated by classical sequencing approaches like the Sanger. Using this novel method to compare SAD and control samples, we have identified new SAD-specific SNVs in genes present in the X chromosome of brain cells.

## Methods

### Characteristics of donors

The characteristics of non-demented controls and SAD-diagnosed donors are summarized in Table 1.

### Brain tissue processing and genomic DNA extraction

Hippocampal and blood tissue samples were extracted and processed as described in^6^. Genomic DNA was extracted from hippocampal tissue samples of donors who had been clinically and neuropathologically confirmed as SAD cases and from control donors with no neurological or neuropathological hallmarks of the disease. Brain tissue samples were obtained from two Spanish brain banks (*Banco de Tejidos* CIEN [BT- CIEN] and *Biobanco del Sistema Sanitario Público de Andalucía*). Donors gave their written informed consent and the tissues were obtained using protocols approved by the ethical committee of the *Banco de Tejidos CIEN [BT- CIEN]* and the *Biobanco del Sistema Sanitario Público de Andalucía*. Our protocols and methods were previously approved by the ethical committee of our center (*Comité de Ética de la Investigación conjunto CNB-CBMSO*, http://www.cnb.csic.es/~cei/). The methods were carried out in accordance with the approved guidelines. DNA was extracted using Qiagen kits and following the manufacturer’s instructions.

### Sample processing for exome sequencing

A Covaris LE220 instrument was used to fragment 3 μg of genomic DNA (from brain and blood) to an average size of 200 bp. Short insert libraries were obtained using the Illumina TruSeq DNA Sample Preparation Kit. Exonic sequences were enriched using NimbleGen Sequence Capture Human Exome 2.1M Array. Paired-end sequences of 91 nucleotides from each end were generated using an Illumina HiSeq 2000 instrument to an average of 50x coverage. Sequences were generated in FastaQ format.

### Bioinformatic analysis

Our analysis was based on the recommended workflows and good practices for variant analysis using the software suite Genome Analysis Tool Kit (GATK). Samples in FastaQ format were aligned to the human reference genome version GRCh37 using the BWA aligner software^25^ with default parameters and were then preprocessed by removing duplicate reads using Picard software (https://broadinstitute.github.io/picard/). Local realignment was performed around insertions and deletions (INDELs) in order to improve SNV calling in these conflictive areas (IndelRealigner from^26^. Base quality scores were then recalibrated using the BaseRecalibrator tool from GATK. Recalibrated samples in BAM format were used to call SNVs and INDELs simultaneously with the HaplotypeCaller algorithm from^26^. At this point, we applied three methods depending on the analysis of interest:

**A**) To obtain all the exonic SNVs from each individual (see Fig. 2A), all files containing raw variants were treated individually with a workflow based on GATK best practices (https://www.broadinstitute.org/gatk/guide/best-practices). Briefly, raw variants in gVCF format were genotyped with GenotypeGVCF from^26^. Before filtration, only SNVs were selected from raw files and separated from INDELs with SelectVariants algorithm from^26^. The files containing raw variants were filtered using the following parameters: coverage: DP > 20, QD < 2.0; FS > 60.0, MQ < 35.0; HaplotypeScore > 13.0; MQRankSum < −12.5, ReadPosRankSum < −8.0 and QUAL > 30. We selected only calls that passed these filters. The variants were then annotated using the dbSNP database version 138^27^, the UCSC human RefGene^26^,^28^, and snpEFF software version 3.6^29^.

**B)** In order to obtain variants present in the exomes of SAD patients, a similar method to that described in A was applied, but in this case comparing each file containing filtered and recalibrated exonic variants from SAD patients with those from control subjects and selecting only those SNVs present in SAD samples but absent in controls (see Fig. 2B). This was achieved using HTSlib/Samtools software^30^.

**C)** An alternative method was also used to compare files containing SNVs and to obtain those variants present only in SAD samples (see Fig. 2C). The difference between this method and the previous one is that the comparison between SAD and control files was done at a previous level, when all the variants had not been filtered (raw variants). Using this approach, we prevented the loss of true SNVs and their appearance as false negatives. As in **B**, we selected only variants present in the SAD individuals but in none of the controls. Having completed this step, we proceeded by filtering, recalibrating, and annotating the SNVs obtained with the same software and parameters as in methods **A** and **B**.

GWAs data for human atxn3l, col4a6 and ube2 nl genes were obtained from http://www.gwascentral.org/.

### Polymerase chain reaction (PCR) amplification and Sanger sequencing

To validate some of the results obtained by Illumina sequencing, we performed Sanger sequencing on PCR-amplified genomic DNA containing the studied loci. The oligonucleotide primers ATX3NL_fw (5′-TGCAGGCTCAAAAATCAAAGGA-3′) and ATX3NL_rv (5′-TCCGGAAACACATCGCAAGA-3′) were used to amplify and then sequence a 539-bp fragment containing the SNV rs4830842 located in *ATXN3L*. The PCR products were electrophoresed in 1% agarose gel stained with Sybr Safe DNA gel stain (Life Technologies) and visualized under UV light. Sequencing was performed by Macrogen Europe (Amsterdam, the Netherlands).

## Additional Information

**How to cite this article**: Gómez-Ramos, A. *et al*. Distinct X-chromosome SNVs from some sporadic AD samples. *Sci. Rep*. **5**, 18012; doi: 10.1038/srep18012 (2015).

## Supplementary Material

Supplementary Information

## Figures and Tables

**Table 1:** Characteristics of the donors of the samples used in this study.

Code	Gender	Age	Diagnose
A1	M	94	Alzheimer
A2	F	85	Alzheimer
A3	F	84	Alzheimer
A4	F	84	Alzheimer
A5	M	78	Alzheimer
C1	M	86	–
C2	M	64	–
C3	M	46	–
C4	F	87	–
C5	M	80	

The table shows the gender, age and diagnose of the donors of the samples that were used in this work.

**Table 2:** Characteristics of the SNVs present in all the samples from sporadic Alzheimer disease patients but not in the non-demented controls.

*Chrom*	*Pos*	*ID*	*Gene name*	*Codon change*	*Aa change*	*GT A1*	*GT A2*	*GT A3*	*GT A4*	*GT A5*
chr1	22206649	rs989994	HSPG2	aAt/aGt	N765S	C/C	C/C	C/C	C/C	C/C
**chr1**	**40923019**	**rs2272994**	**ZNF643**	**atG/atA**	**M46I**	**G/A**	**G/A**	**G/A**	**A/A**	**A/A**
**chr1**	**53320274**	**rs480299**	**ZYG11A**	**gaG/gaT**	**E76D**	**T/T**	**G/T**	**T/T**	**T/T**	**T/T**
**chr1**	**150679033**	**rs1336900**	**HORMAD1**	**aCa/aTa**	**T187I**	**G/A**	**G/A**	**G/A**	**G/A**	**G/A**
**chr1**	**150727539**	**rs2230061**	**CTSS**	**Cgg/Tgg**	**R113W**	**G/A**	**G/A**	**G/A**	**G/A**	**G/A**
**chr1**	**150808889**	**rs2228099**	**ARNT**	**gtG/gtC**	**V173**	**C/G**	**C/G**	**C/G**	**C/G**	**C/G**
chr1	201047062	rs4915476	CACNA1S	Ctg/Ttg	L522	G/A	G/A	A/A	G/A	G/A
chr1	201047075	rs4915477	CACNA1S	ggT/ggC	G517	A/G	G/G	G/G	G/G	G/G
**chr1**	**228402508**	**rs2776853**	**OBSCN**	**Ctg/Ttg**	**L513**	**C/T**	**C/T**	**C/T**	**C/T**	**C/T**
chr1	247921100	rs1552812	OR1C1	ggA/ggC	G203	T/G	G/G	G/G	G/G	T/G
**chr2**	**72361960**	**rs2241057**	**CYP26B1**	**tTg/tCg**	**L189S**	**A/G**	**A/G**	**A/G**	**A/G**	**A/G**
**chr2**	**100916315**	**rs11123823**	**LONRF2**	**ggT/ggC**	**G134**	**G/G**	**A/G**	**A/G**	**G/G**	**A/G**
**chr2**	**100917109**	**rs13006224**	**LONRF2**	**tcG/tcA**	**S111**	**T/T**	**C/T**	**C/T**	**T/T**	**C/T**
**chr2**	**101010082**	**rs3748930**	**CHST10**	**acC/acG**	**T232**	**C/C**	**G/C**	**G/C**	**C/C**	**G/C**
**chr2**	**101646144**	**rs3739015**	**TBC1D8**	**tcC/tcT**	**S662**	**G/A**	**G/A**	**G/A**	**G/A**	**G/A**
chr3	33138544	rs7614776	GLB1	Ttg/Ctg	L12	G/G	G/G	A/G	G/G	G/G
chr3	119133183	rs3732413	ARHGAP31	Ggc/Agc	G803S	G/A	A/A	A/A	A/A	A/A
**chr4**	**76489582**	**rs2306174**	**C4orf26**	**Gtt/Att**	**V124I**	**G/A**	**G/A**	**G/A**	**G/A**	**G/A**
**chr4**	**79387442**	**rs7660664**	**FRAS1**	**caC/caT**	**H2370**	**C/T**	**C/T**	**C/T**	**C/T**	**C/T**
**chr4**	**159881479**	**rs9784569**	**C4orf45**	**caA/caG**	**Q105**	**C/C**	**C/C**	**C/C**	**T/C**	**T/C**
chr6	150067675	rs10872646	NUP43	gTa/gCa	V47A	A/G	A/G	A/G	A/G	A/G
**chr6**	**160952838**	**rs3124784**	**LPA**	**Cgc/Tgc**	**R2016C**	**G/A**	**G/A**	**G/A**	**G/A**	**G/A**
chr7	100731829	rs6948536	TRIM56	gcT/gcC	A412	T/C	C/C	T/C	C/C	T/C
**chr7**	**150439500**	**rs759011**	**GIMAP5**	**gcC/gcT**	**A127**	**C/T**	**C/T**	**T/T**	**C/T**	**C/T**
chr8	144941181	rs7839934	EPPK1	Ctg/Gtg	L2081V	G/C	G/C	G/C	C/C	G/C
chr9	34729452	rs10115191	RP11-195F19.10	NA	NA	A/G	A/G	A/G	A/G	A/G
chr9	125239253	rs1962091	OR1J1	aAc/aGc	N318S	C/C	C/C	C/C	T/C	T/C
chr10	50532683	rs7921186	C10orf71	tTc/tCc	F698S	C/C	C/C	C/C	C/C	C/C
chr10	70405855	rs3998860	TET1	atA/atG	I1123M	G/G	G/G	G/G	G/G	G/G
**chr10**	**91007360**	**rs1051338**	**LIPA**	**Acc/Ccc**	**T16P**	**T/G**	**T/G**	**T/G**	**T/G**	**T/G**
**chr11**	**4790396**	**rs17324609**	**OR51F1**	**gCt/gTt**	**A251V**	**A/A**	**G/A**	**G/A**	**A/A**	**A/A**
**chr11**	**4790482**	**rs12792898**	**OR51F1**	**ttA/ttG**	**L222**	**C/C**	**T/C**	**T/C**	**C/C**	**C/C**
**chr11**	**4790575**	**rs12788102**	**OR51F1**	**tgT/tgC**	**C191**	**G/G**	**A/G**	**A/G**	**G/G**	**G/G**
chr11	4824878	rs2053116	OR52R1	Tcc/Gcc	S245A	C/C	A/C	C/C	C/C	C/C
**chr11**	**4825349**	**rs17327254**	**OR52R1**	**Ttc/Ctc**	**F167L**	**G/G**	**A/G**	**A/G**	**G/G**	**G/G**
**chr11**	**4842866**	**rs35003053**	**OR51F2**	**gAc/gGc**	**D84G**	**G/G**	**A/G**	**A/G**	**G/G**	**G/G**
**chr11**	**4870284**	**rs35918613**	**OR51S1**	**aCc/aGc**	**T52S**	**C/C**	**G/C**	**G/C**	**C/C**	**C/C**
**chr11**	**4944892**	**rs34583466**	**OR51G1**	**acC/acG**	**T226**	**C/C**	**G/C**	**G/C**	**C/C**	**C/C**
**chr11**	**4944986**	**rs12796015**	**OR51G1**	**aTt/aCt**	**I195T**	**G/G**	**A/G**	**A/G**	**G/G**	**G/G**
**chr11**	**4945199**	**rs34742470**	**OR51G1**	**cGc/cAc**	**R124H**	**T/T**	**C/T**	**C/T**	**T/T**	**T/T**
chr11	7949791	rs7933807	OR10A6	gTt/gGt	V140G	A/C	A/C	A/C	C/C	C/C
**chr11**	**12525925**	**rs11547363**	**PARVA**	**cgC/cgT**	**R149**	**C/T**	**C/T**	**C/T**	**C/T**	**C/T**
**chr11**	**18422487**	**rs61736803**	**LDHA**	**atC/atA**	**I116**	**C/A**	**C/A**	**C/A**	**C/A**	**C/A**
**chr11**	**44940828**	**rs2291334**	**TSPAN18**	**Gtc/Atc**	**V133I**	**G/A**	**G/A**	**G/A**	**G/A**	**G/A**
chr11	55872876	rs2512961	OR8H2	Cat/Tat	H120Y	T/T	T/T	T/T	T/T	T/T
**chr11**	**56510623**	**rs513873**	**OR9G4**	**gTa/gCa**	**V222A**	**G/G**	**G/G**	**A/G**	**A/G**	**A/G**
**chr11**	**56510694**	**rs1397053**	**OR9G4**	**ccA/ccG**	**P198**	**T/C**	**T/C**	**T/C**	**T/C**	**T/C**
chr11	74862391	rs1944612	SLCO2B1	NA	NA	G/G	G/G	G/G	G/G	G/G
**chr11**	**108175462**	**rs1801516**	**ATM**	**Gat/Aat**	**D1853N**	**G/A**	**G/A**	**G/A**	**G/A**	**G/A**
chr11	117266312	rs2305830	CEP164	aCc/aGc	T962S	C/G	C/G	C/G	C/G	C/G
chr12	109693982	rs3742023	ACACB	caC/caT	H1299	T/T	C/T	C/T	C/T	C/T
chr13	46946157	rs1408184	KIAA0226L	Ggg/Agg	G152R	T/T	C/T	C/T	C/T	C/T
**chr13**	**49776080**	**rs9316430**	**FNDC3A**	**aaA/aaG**	**K1044**	**A/G**	**A/G**	**A/G**	**A/G**	**A/G**
chr14	64637147	rs7161192	SYNE2	ctC/ctA	L2119	C/A	A/A	C/A	C/A	C/A
**chr15**	**58838038**	**rs6084**	**LIPC**	**acC/acG**	**T163**	**C/G**	**G/G**	**C/G**	**G/G**	**C/G**
chr15	78390909	rs12593575	SH2D7	Cgg/Tgg	R206W	C/T	C/T	C/T	C/T	C/T
chr16	2812890	rs2240141	SRRM2	aaA/aaG	K39	G/G	G/G	A/G	G/G	G/G
chr16	3490922	rs2270494	ZNF597	ctC/ctG	L15	G/C	C/C	G/C	C/C	G/C
**chr18**	**59936142**	**rs17645999**	**KIAA1468**	**gtC/gtT**	**V907**	**T/T**	**C/T**	**T/T**	**C/T**	**C/T**
chr19	2917612	rs10410539	ZNF57	acT/acC	T299	C/C	C/C	T/C	T/C	T/C
chr19	18679379	rs7648	C19orf50	Cct/Gct	P157A	C/G	G/G	G/G	G/G	G/G
**chr19**	**20748522**	**rs12979592**	**ZNF737**	**NA**	**NA**	**C/A**	**C/A**	**C/A**	**C/A**	**C/A**
chr19	34959979	rs7259160	UBA2	tcA/tcG	S496	A/G	G/G	G/G	A/G	G/G
**chr20**	**13134768**	**rs6078938**	**SPTLC3**	**taT/taC**	**Y466**	**C/C**	**T/C**	**T/C**	**T/C**	**T/C**
chr20	55108617	rs3209183	C20orf107	Cag/Aag	Q74K	A/A	A/A	C/A	C/A	C/A
**chr21**	**33887131**	**rs1129157**	**C21orf63**	**ccG/ccA**	**P271**	**G/A**	**G/A**	**G/A**	**G/A**	**A/A**
**chr22**	**18209920**	**rs9306198**	**BCL2L13**	**Cct/Tct**	**P198S**	**T/T**	**C/T**	**C/T**	**C/T**	**C/T**
chr22	32554985	rs5998267	C22orf42	cTg/cCg	L73P	G/G	A/G	A/G	A/G	A/G
**chrX**	**13337059**	**rs4830842**	**ATXN3L**	**gGc/gAc**	**G332D**	**C/T**	**C/T**	**C/T**	**T/T**	**C/T**
chrX	55514818	rs3126255	USP51	gaG/gaA	E185	C/T	C/T	C/T	T/T	T/T
chrX	70146398	rs4360450	SLC7A3	agT/agC	S533	A/G	G/G	A/G	G/G	G/G
chrX	74494470	rs4892396	UPRT	cgT/cgG	R127	G/G	G/G	G/G	G/G	G/G
chrX	85219021	rs10217950	CHM	gcA/gcG	A117	T/C	T/C	T/C	C/C	C/C
chrX	88008807	rs5984611	CPXCR1	cGt/cAt	R131H	G/A	G/A	G/A	G/A	G/A
chrX	105153001	rs209372	NRK	aaA/aaG	K456	G/G	G/G	G/G	G/G	G/G
**chrX**	**107417730**	**rs5973851**	**COL4A6**	**ggC/ggT**	**G1026**	**G/A**	**G/A**	**G/A**	**G/A**	**G/A**
chrX	114425400	rs12857270	RBMXL3	Gga/Aga	G466R	A/A	G/A	A/A	A/A	A/A
chrX	117700141	rs2286977	DOCK11	gcA/gcG	A289	A/G	A/G	G/G	A/G	A/G
chrX	118604436	rs12390	SLC25A5	acT/acC	T233	C/C	C/C	T/C	T/C	C/C
chrX	118699320	rs5910616	CXorf56	NA	NA	G/G	G/G	A/G	G/G	G/G
chrX	133379551	rs2428577	CCDC160	Cta/Tta	L241	T/T	T/T	T/T	T/T	T/T
**chrX**	**142967468**	**rs237520**	**UBE2NL**	**tTa/tGa**	L89*	**T/G**	**T/G**	**T/G**	**G/G**	**G/G**
chrX	144904882	rs2748588	SLITRK2	ccT/ccC	P313	T/C	C/C	C/C	C/C	C/C
chrX	153633359	rs1130929	DNASE1L1	ccC/ccG	P67	C/C	C/C	C/C	C/C	C/C

This table shows the position in genome, ID in dbSNP^26^, name of the gene in which the SNVs are located, codon and amino acid changes (if any), and the genotypes (GT) for the SNVs specific to sporadic Alzheimer disease found in the five exomes analyzed. All the SNVs in the table (84) were obtained by method B. Those shown in bold (42 SNVs, included in those obtained by method B) were obtained by method C.
