# Supplementary material for: Distinct X-chromosome SNVs from some sporadic AD samples
Source: Sci Rep. 2015 Dec 9;5:18012. doi: 10.1038/srep18012 (PMC4673451; doi:10.1038/srep18012)
Supplement: Supplementary Information [file srep18012-s1.pdf]

Supplementary Material

Distinct X-chromosome SNVs from some sporadic AD samples

Gómez-Ramos A, Podlesniy P, Soriano E and Avila J

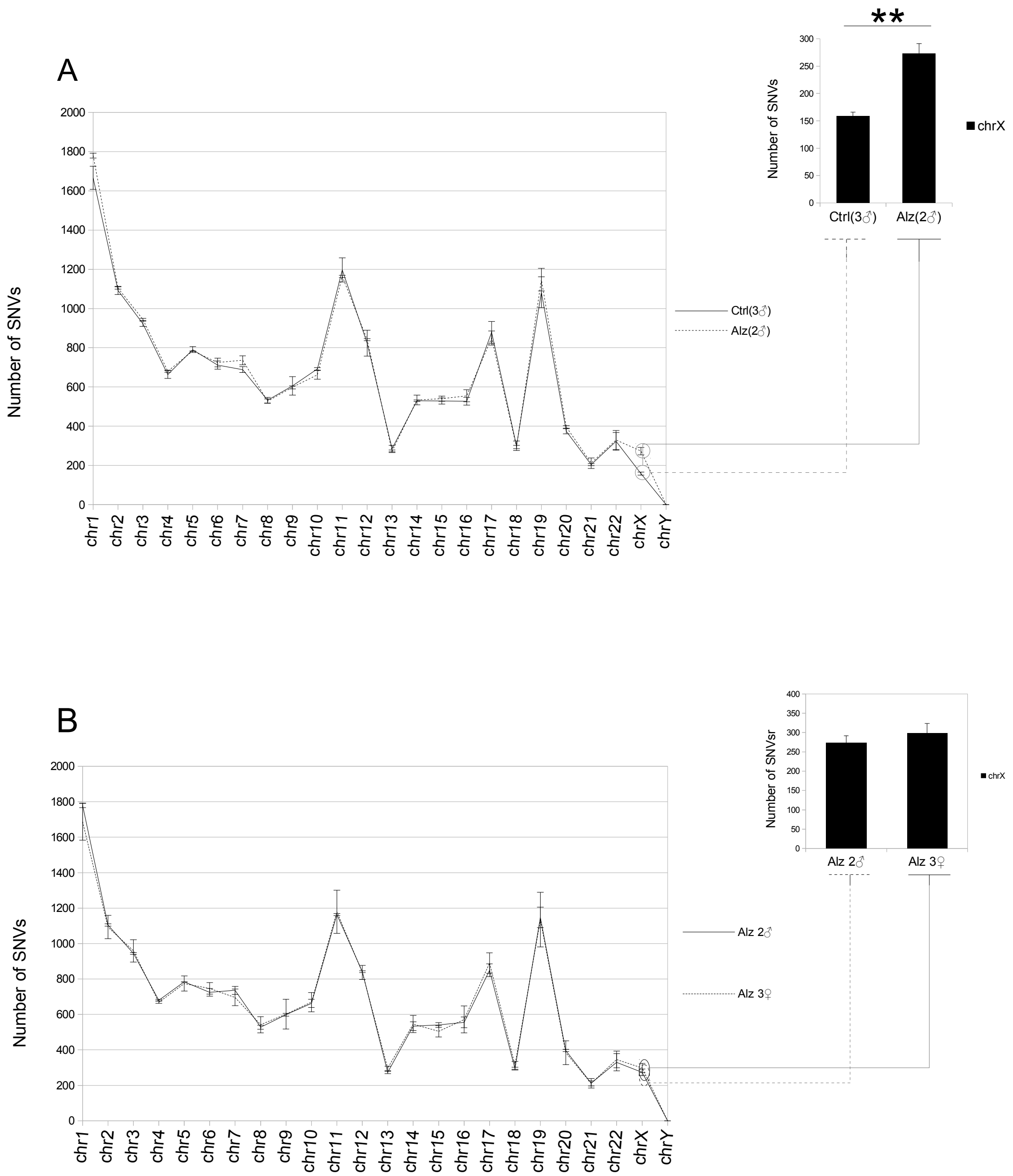
A
B
Number of SNVs
Number of SNVsr
**
chrX
Ctrl(3♂)
Alz(2♂)
Alz 2♂
Alz 3♀
chr1
chr2
chr3
chr4
chr5
chr6
chr7
chr8
chr9
chr10
chr11
chr12
chr13
chr14
chr15
chr16
chr17
chr18
chr19
chr20
chr21
chr22
chrX
chrY

Suppl. fig 1

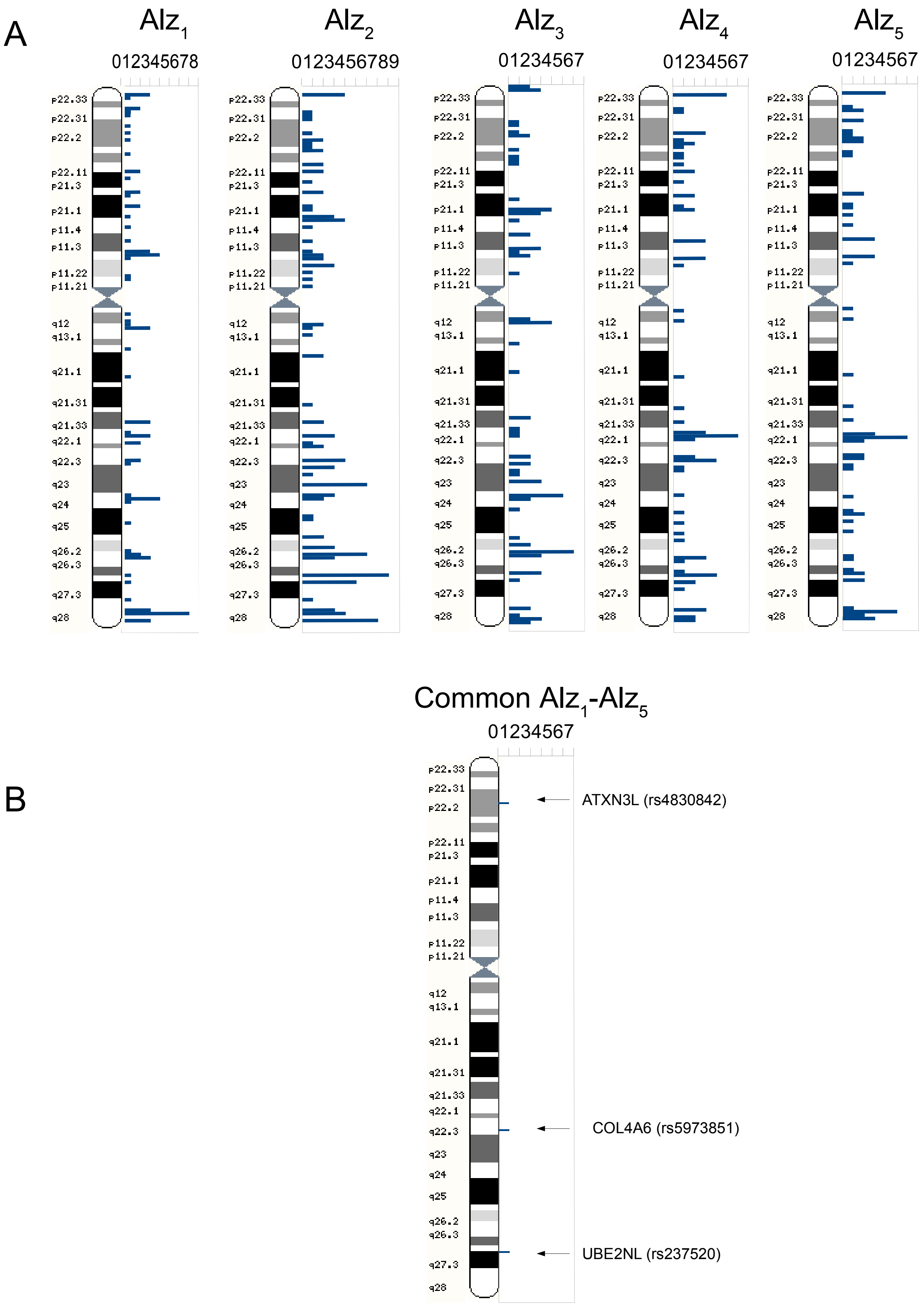
A
Alz1
Alz2
Alz3
Alz4
Alz5
012345678
0123456789
01234567
p22.33
p22.31
p22.2
p22.11
p21.3
p21.1
p11.4
p11.3
p11.22
p11.21
q12
q13.1
q21.1
q21.31
q21.33
q22.1
q22.3
q23
q24
q25
q26.2
q26.3
q27.3
q28
B
Common Alz1-Alz5
ATXN3L (rs4830842)
COL4A6 (rs5973851)
UBE2NL (rs237520)

Suppl. fig 2

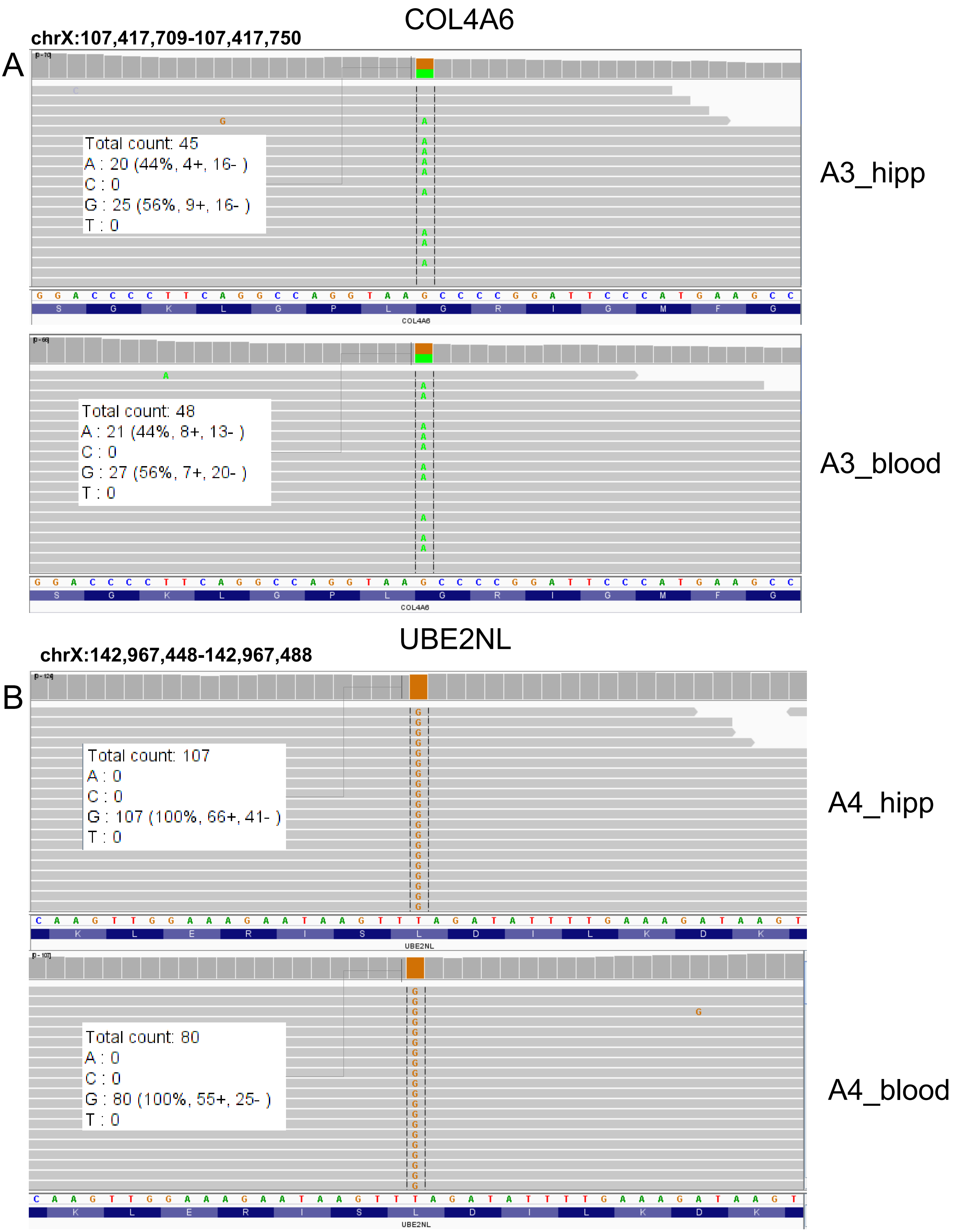

Supp figure 3

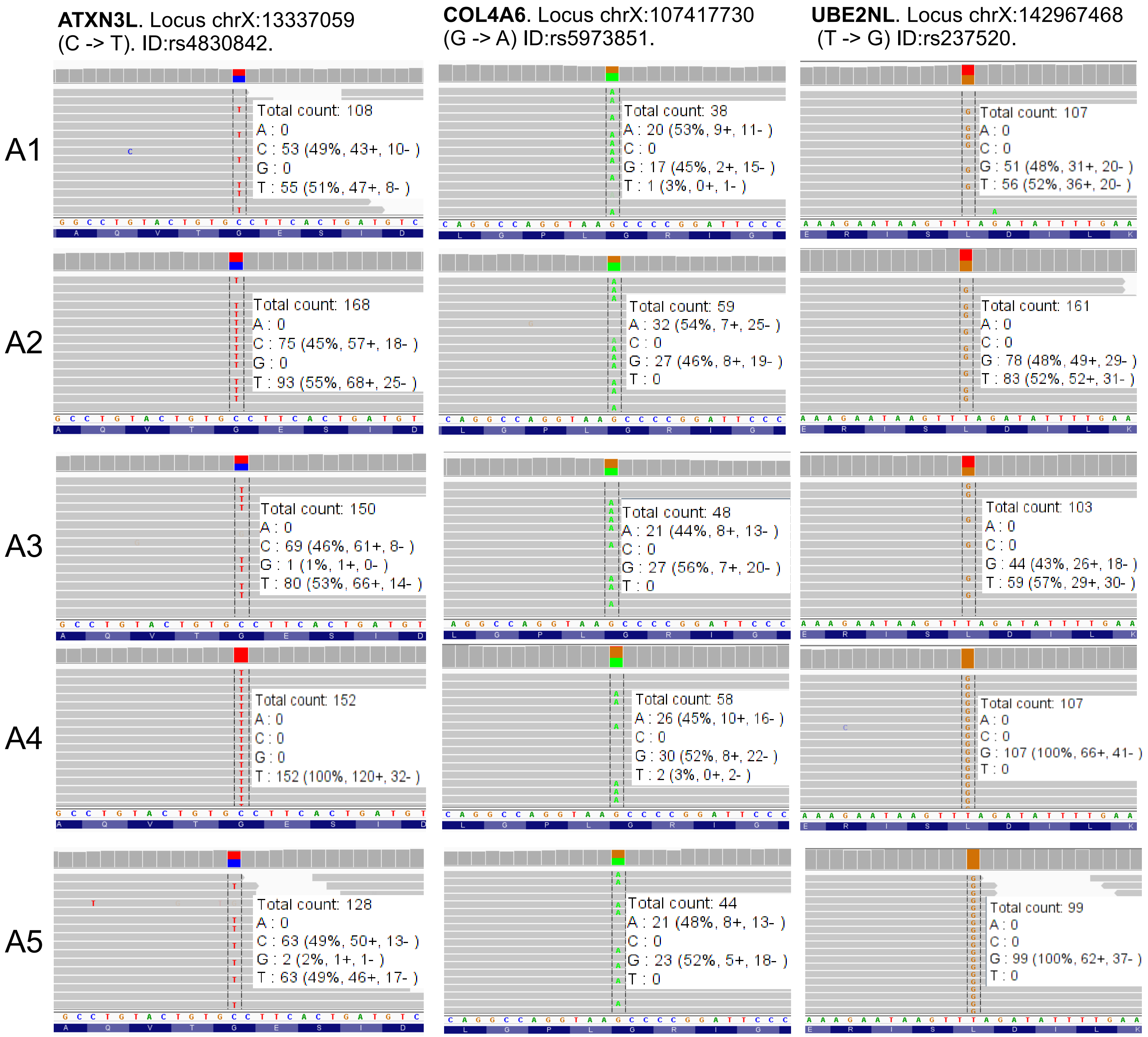

Supp figure 4A

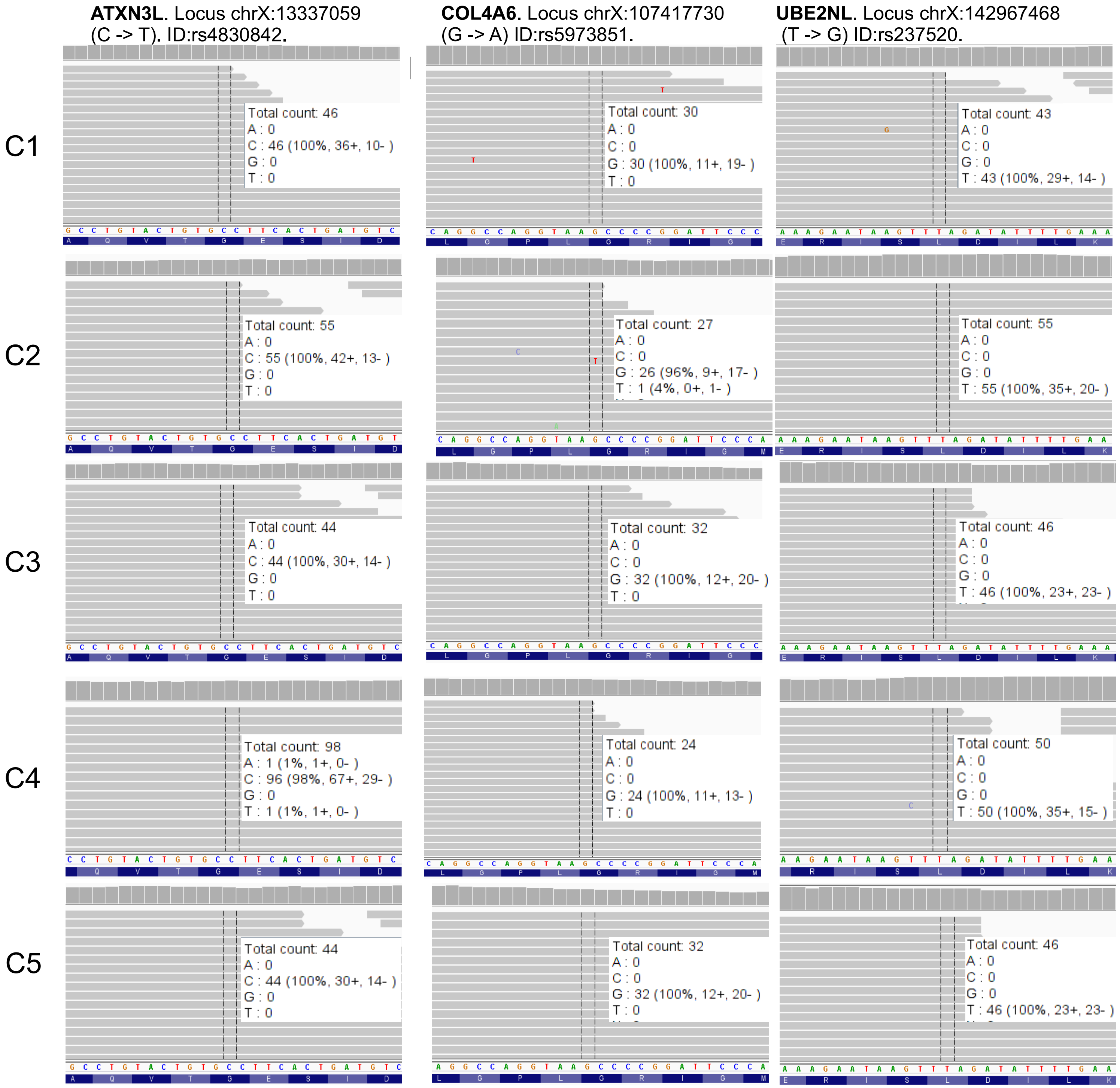

Supp figure 4B

Chr	*Pos*	*ID (dbSNP)*	*Gene name*	*Codon change*	*Aa change*	Control genotypes					Alzheimer genotypes				
						C1	C2	C3	C4	C5	A1	A2	A3	A4	A5
X	13337059	rs4830842	ATXN3L	gGc/gAc	G332D	C/C	C/C	C/C	C/C	C/C	C/T	C/T	C/T	T/T	C/T
X	107417730	rs5973851	COL4A6	ggC/ggT	G1026	G/G	G/G	G/G	G/G	G/G	G/A	G/A	G/A	G/A	G/A
X	142967468	rs237520	UBE2NL	tTa/tGa	L89*	T/T	T/T	T/T	T/T	T/T	T/G	T/G	T/G	G/G	G/G

Supp table1

Supplementary Figure 1: The difference in the number of SNVs found in the X chromosome is not a consequence of donor gender.

A) Graphic showing the average number of SNVs per chromosome for all the male samples and grouped by diagnosis (sporadic Alzheimer disease (SAD) or non-demented controls). The result is very similar to that shown in Figure 2B, and there is a significant difference in the number of SNVs for the X chromosome between SAD and control samples (see inset).

B) As in **A)**, the number of SNVs per chromosome is shown in this plot; however, in this case, the samples were grouped by gender, and only the samples from SAD patients are included. No significant difference between the number of SNVs in the X chromosome (inset) is observed.

Error bars show the standard deviation in each case and double asterisk is statistically significant ($P < 0.001$) compared with control cases.

Supplementary Figure 2: Distribution of the number of SNVs specific to sporadic Alzheimer disease along the X chromosome as determined by method C.

A) This figure shows the distribution of the number of SAD-specific SNVs (present in samples from patients with sporadic Alzheimer disease (SAD) but not in the non-demented controls) along the X chromosome. The results correspond to those represented in Venn diagrams in Figure 3A obtained by method C for the X chromosome and named Alz 1-Alz5. Each thin blue bar represents the number of SAD-specific SNVs found every 106 bp.

B) As a **A)**, the distribution of the number of SAD-specific SNVs along the X chromosome, but in this case showing only those specific and common to all SAD samples.

The SNV distribution pattern along the X chromosome is random

Supplementary Figure 3: Analysis of Alzheimer-specific SNVs found in chromosome X in non-neuronal tissues.

The figure shows the alignment of the processed reads (see methods) for two of the SNVs characterized in this work found in chromosome X in two of the Alzheimer cases (A3 and A4) described in this study. We compared hippocampal with blood exome in two different loci (COL4A6 and UBE2NL) located in chromosome X, described in table 2.

A) Presence of a SNV (ID: rs4830842, located in position X:107417730 in gene COL4A6, see table 2) in the Alzheimer diagnosed individual A3 (see table 1) in hippocampus (A3_hipp) and blood (A3_blood) samples.

B) The SNV rs237520, in position X:142967468 in gene UBE2NL (see table 2) is shown in hippocampal (A4_hipp) an blood (A4_blood) exomes.

Results show a very similar pattern of coverage for both SNVs, indicating the presence of the same SNV and genotype (heterozigous in **A** and homozigous in **B)** in both tissues in each case. This result suggest a common provenience of the SNVs for both tissues (hippocampus and blood), having probably a germline origin in these two cases.

Supplementary Figure 4:

A) Figure shows the alignment of the sequenced reads for all the Alzheimer samples used in this work (see characteristics in methods) in three of the loci located in chromosome X (A, ATXN3L; B, COL4A6 and C, UBE2NL) in which Alzheimer-specific SNVs were found using the method C described in the manuscript. All of them have the described SNV.

B) As in A, the alignments for these three Alzheimer-specific SNV are shown for the non-demented control cases. In order to confirm this genotype, two additional non-demented controls have been added C4 (female, age 87) and C5 (male, age 80) . As it can see, none of the control samples have the SNVs, being homozygous for the reference alele.

Supplementary Table 1:

This table shows the position in genome, ID (according to dbSNP database), name of the gene in which the SNVs are located, codon and amino acid changes (if any), and the genotypes for the Alzheimer-specific SNVs found in chromosome X.
